# Reply to the Comment on: Subrat Khanal et al. The Repertoire of Adenovirus in Human Disease: The Innocuous to the Deadly. *Biomedicines* 2018, *6*, 30

**DOI:** 10.3390/biomedicines7010010

**Published:** 2019-02-02

**Authors:** Subrat Khanal, Pranita Ghimire, Amit S. Dhamoon

**Affiliations:** Department of Medicine, State University of New York, Upstate Medical University, Syracuse, NY 13210, USA; ghimirep@upstate.edu (P.G.); dhamoona@upstate.edu (A.S.D.)

We would like to thank Dr. Atkinson for his comments on our review article on the manifestations of adenoviral infections in humans. We also congratulate him on his extensive work on adenovirus and obesity. Obesity is a complex multifactorial disease influenced by genetics, lifestyle, metabolic factors, culture, and the environment. As discussed in Dr. Atkinson’s comment, one of the many factors that influence one’s propensity for obesity is exposure to adenovirus 36. The concept that an adenoviral vaccine can mitigate this public health epidemic is intriguing and thought-provoking. Although data suggest that there is a relationship between adenoviral infection and obesity, the data do not indicate that the current obesity epidemic is primarily attributable to the exposure of adenovirus. 

Per a 2014 study designed to assess adenoviral infection status and subsequent weight gain, the adult military personnel that were studied did not have a subsequent increase in BMI associated with adenoviral infection [1]. Given the multifactorial nature of the disease, environmental factors including high caloric intake and relatively sedentary lifestyles have appropriately garnered the most attention as risk factors [2,3]. Besides this, a myriad of comorbidities including diabetes mellitus, hypertension, dyslipidemia, medications (such as antidepressants, antipsychotics, and antihyperglycemics), endocrine disorders, hypothalamic syndromes, sleep and feeding disorders have been associated with obesity. Genetic factors such as single gene disorders in the expression of proopiomelanocortin [4] and leptin [5] have been studied extensively as risk factors for obesity. Ghrelin [6], a gut hormone, that increases appetite and inhibitors like Glucagon-Like-Peptide (GLP) and Cholecystokinin are also being studied [7]. A possible explanation towards the association between obesity and adenoviral infections could be increased susceptibility of obese individuals to various viral infections. Multiple studies have also been conducted in this topic and have shown an increased risk for all infections overall [8,9,10], viral infections like influenza [11], surgical site infections [11], nosocomial infections, as well as serious complications of common infections [10]. This is, however, yet to be fully explained and warrants further studies of metabolism and immune response.
